
JOURNAL INFORMATION
==============================
NLM Title Abbreviation: Wirtschaftsdienst
Journal ID: Wirtschaftsdienst
NLM Journal ID: 101086612

Wirtschaftsdienst (Hamburg, Germany : 1949)

ISSN: 0043-6275

ARTICLE INFORMATION
==============================
PMCID: PMC8218272
PMID: 34176984
DOI: 10.1007/s10273-021-2934-1
Article ID: 2934
Article version: 1
Subjects: Zeitgespräch

Bidens Fiskalpolitik — ein Vorbild für Deutschland?

Bachmann Rüdiger rbachman@nd.edu

grid.131063.6 0000 0001 2168 0066 Department of Economics, University of Notre Dame, 3026 Nanovic Hall, Notre Dame, IN 46556 USA
Electronic publication date: 2021 Jun 22
Print publication date: 2021
Volume: 101
Issue: 6
First page: 414
Last page: 417
Copyright: © Der/die Autor:in(nen) 2021
License: Open Access: Dieser Artikel wird unter der Creative Commons Namensnennung 4.0 International Lizenz veröffentlicht (https://creativecommons.org/licenses/by/4.0/deed.de).
Open Access wird durch die ZBW — Leibniz-Informationszentrum Wirtschaft gefördert.
License URL: https://creativecommons.org/licenses/by/4.0/

issue-copyright-statement: © The Author(s) 2021

==============================

Die US-amerikanische Regierung hat in relativ kurzer Folge drei Fiskalprogramme zur Konjunkturstimulierung verabschiedet: am 27. März 2020, bereits zu einem frühen Zeitpunkt in der COVID-19-Pandemie, den CARES (Coronavirus Aid, Relief, and Economic Security) Act mit einem Umfang von 2,2 Billionen US-$, am 27. Dezember 2020 den Consolidated Appropriations Act 2021 im Umfang von 0,9 Billionen US-$, und schließlich dann am 11. März 2021 nach der Amtseinführung Präsident Bidens den American Rescue Plan Act im Umfang von 1,9 Billionen US-$. Zusammen entspricht dies einem fiskalischen Impuls von knapp einem Viertel des US-amerikanischen Bruttoinlandsprodukts (BIP). Darüber hinaus diskutieren Präsident Biden und die Demokraten im Kongress zurzeit ein Infrastrukturpaket, den American Jobs Plan, sowie ein Familien- und Bildungspaket, den American Families Plan. Deren Umfänge betragen weitere Billionen US-Dollar und beinhalten nicht nur klassische Infrastrukturvorhaben, sondern auch Programme zum Klimaschutz und zum Ausbau der Care Economy sowie familien- und bildungspolitische Maßnahmen wie einen Kindergartenanspruch für alle und Kündigungsschutz im Krankheitsfall. Die beiden noch ausstehenden Ausgabenpakete sollen durch höhere Unternehmensteuern, eine höhere Mindeststeuer und verschiedene steuerpolitische Maßnahmen im oberen Einkommenssegment bei der Bundeseinkommensteuer sowie durch zusätzliche Staatsschulden finanziert werden. Auf die Realisierungschancen dieser Pakete, die ja für die Frage wichtig sind, inwieweit die Biden-Politik Vorbild für die Fiskalpolitik in Deutschland sein kann, wird später einzugehen sein.

Abbildung 1 visualisiert die enorme fiskalische Expansion in den USA während der COVID-19-Pandemie. Selbst in der Großen Rezession 2008/2009 tat man nichts Vergleichbares. Abbildung 1 zeigt auch, dass die US-amerikanischen Haushalte im Durchschnitt in einem bisher nicht dagewesenen Ausmaß verfügbares Einkommen aufbauten, und zwar parallel zur fiskalischen Expansion. Trotzdem zeichnet sich in den USA auch eine sich verfestigende Langzeitarbeitslosigkeit sowie eine erhöhte Nahrungsmittelunsicherheit bei den ärmeren Haushalten ab, und das diente letztlich der politischen Rechtfertigung des American Rescue Plan Act durch die Biden Administration.

Für die Beurteilung der ökonomischen Folgen des American Rescue Plan Act sowie die Abschätzung der konjunkturellen Wirkungen der beiden noch nicht verabschiedeten Programme illustriert Abbildung 1 darüber hinaus die *erste* gegenwärtig noch offene Grundfrage:

1. Wie werden die Haushalte mit ihrem zusätzlichen verfügbaren Einkommen umgehen?

Handelt es sich gewissermaßen um eine „Kriegskasse“, die in kurzer Zeit für einen postpandemischen Konsumrausch ausgegeben werden wird? Oder wird das zusätzliche verfügbare Einkommen zur Reparatur der Haushaltsbilanzen verwendet, also letztlich ins langfristige Nettovermögen der Haushalte eingehen und erst allmählich nachfragewirksam werden? Wir kennen die Antwort noch nicht. Tritt eher das erste Szenario ein, wird für einige Zeit mit kräftiger Inflation, höheren Nominalzinsen und mit Angebotsrestriktionen zu rechnen sein. Gerade letztere stellen dann aber auch ein Problem für den American Jobs Plan und den American Families Plan dar, die zwar nicht konjunkturell, sondern langfristig motiviert sind, aber eben dann auf eine ohnehin bereits ausgelastete US-amerikanische Wirtschaft mit brummender Konjunktur träfen.

Die *zweite* noch offene Grundfrage ist:

2. Wie schnell wird die Outputlücke bzw. die Arbeitslosigkeit in den USA abgebaut werden?

Abbildung 2 zeigt die Entwicklung der Outputlücke[1] sowie der Arbeitslosenrate während der Coronakrise im

1 Outputlückenschätzungen sind nicht unproblematisch und werden immer wieder kritisiert. Hier geht es aber um den Unterschied in der Outputlückenentwicklung nach der Großen Rezession gegenüber derjenigen während der COVID-19-Krise.

Abbildung 1

**Nominale Gesamtausgaben des Staates und reales verfügbares Einkommen pro Kopf in den USA**

Vierteljährlich, saisonbereinigte Jahresraten

Anmerkungen: US-Rezessionen sind grau hinterlegt; das jüngste Enddatum ist unbekannt.

Quelle: FRED.

Abbildung 2

**Realer Potenzialoutput, reales BIP und Arbeitslosenrate in den USA**

Vierteljährlich, saisonbereinigte Jahresraten

Anmerkungen: US-Rezessionen sind grau hinterlegt; das jüngste Enddatum ist unbekannt.

Quelle: FRED.

Vergleich zu den beiden vorherigen Rezessionen. Damals wurde die Outputlücke bzw. Arbeitslosenrate nur sehr langsam abgebaut, ein typisches konjunkturelles Muster für die USA. Ganz anders die Entwicklung während der Corona-Krise: Die Ausschläge waren deutlich größer als selbst in der Großen Rezession, aber der Abbau verlief – bisher – relativ schnell. Wenn sich die Dynamik so weiter entwickelt, zeichnen sich auch hier inflationäre Entwicklungen aufgrund von Angebotsrestriktionen ab.

Die *dritte* offene Grundfrage, oder vielmehr offene Flanke der Bidenschen Ausgabenpläne, ist die Entwicklung der Staatsschulden insgesamt:

3. Wie werden sich Defizit und Staatsschuldenstand in den nächsten Jahren entwickeln?

Abbildung 3 zeigt die historische und prognostizierte Entwicklung der Staatsschuldenquote der USA unter der Annahme, dass alle aktuellen Gesetze in Kraft bleiben. Die bisher nicht verabschiedeten Bidenschen Ausgabenpläne sind also noch gar nicht Teil dieser Prognose. Ein Großteil der gezeigten explosiven Dynamik ergibt sich dabei aus dem prognostizierten Zinswachstum.

Angesichts dieser Entwicklungen darf man schon etwas nervös in Bezug auf die Nachhaltigkeit der US-amerikanischen Fiskalpolitik werden. Und in der Tat zeigen die Daten bereits einen, wenn auch nicht dramatischen Anstieg sowohl der Nominalzinsen für amerikanische Staatsanleihen am längeren Ende als auch der längerfristigen In-

Abbildung 3

**Entwicklung und Prognose der Staatsschuldenquote der USA**

Anteil am BIP in %

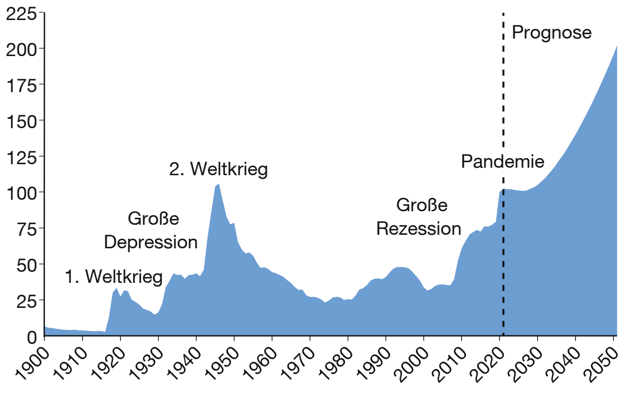

Anmerkungen: Wachsende Defizite werden die Staatsverschuldung in den nächsten 30 Jahren voraussichtlich auf ein noch nie dagewesenes Niveau treiben. Bis 2051 wird die Verschuldung voraussichtlich mehr als 200 % des BIP erreichen.

Quelle: Congressional Budget Office (2021).

flationserwartungen[2] der amerikanischen Haushalte und Finanzmärkte. Natürlich gibt es gute, direkt wachstumsrelevante Gründe für den American Jobs Plan und den American Families Plan: Die US-amerikanische Infrastruktur ist – übrigens mit großem politischen Konsens – erneuerungsbedürftig; die US-amerikanische Wirtschaft muss dekarbonisiert werden, wodurch Arbeitsplätze im Verarbeitenden Gewerbe und Baugewerbe entstehen werden; der Breitbandausbau ist wachstumsfördernd, vor allem wenn er zu effizienterer Telearbeit führt; und selbst die geplanten familien- und bildungspolitischen Maßnahmen dürften das Arbeitsangebot vor allem von Frauen langfristig erhöhen. Dennoch ist es zum heutigen Zeitpunkt offen, ob die angebotsseitigen Effekte mit denen auf der Nachfrageseite Schritt halten können, sodass es zu keiner inflationären Überhitzung der US-Wirtschaft käme, und ob die Wachstumseffekte insgesamt so stark sein werden, dass die USA aus ihren Staatsschulden herauswachsen können.

Hinzu kommt die politische Unsicherheit in den USA. Sowohl von der politischen als auch kommentatorischen Linken in Deutschland wird Biden wegen seiner Politik und der Geschwindigkeit, mit der er diese vorantreibt, hoch gelobt und als Vorbild für die (Fiskal-)Politik in Deutschland angesehen. Dabei wird aber übersehen, dass lediglich der sehr auf konjunkturelle Wirkungen abzielende American Rescue Plan bereits Gesetz ist, die anderen, eher auf das längerfristige Wachstum zielenden Pläne aber noch nicht verabschiedet wurden, und dass sich zurzeit auch kein gutes politisches Schicksal für sie abzuzeichnen scheint. Das gilt auch und gerade für die Steuererhöhungen bei Unternehmen und hohen Einkommensbeziehern. Ohne allzu sehr in die Details der recht komplizierten parlamentarischen Regeln des US-Senats zu gehen, muss man feststellen, dass Biden faktisch über keine belastbare parlamentarische Mehrheit für seine Vorhaben verfügt. Die Mehrheit im Repräsentantenhaus ist schon jetzt recht dünn und im Herbst 2022 in den dann anstehenden Midtermwahlen aufgrund der von den Republikanern in den Bundesstaaten dominierten Neugliederung der Wahlbezirke eminent gefährdet. Im Senat hat Biden nur deshalb eine Mehrheit, weil bei der existierenden Stimmengleichheit die demokratische Vizepräsidentin den Ausschlag gibt. Und selbst diese Mehrheit gilt für die meisten Gesetze, die mit einer Mehrheit von 60 % verabschiedet werden müssen, nicht. Zwar gibt es – und hier wird es einigermaßen kompliziert – die Ausnahme, dass auch eine 50-plus-1-Mehrheit für budgetrelevante Gesetze genügt, die sogenannte *Reconciliation*, aber diese Regel darf 2021 nur zweimal angewendet werden. Der American Rescue Plan hat schon eine Anwendung verbraucht. Hinzu kommt, dass Präsident Biden selbst für diese Sonderregel alle 50 demokratischen Mitglieder im Senat braucht,[3] oder einige Mitglieder der Republikaner umstimmen muss. Das ist angesichts der komplizierten Wiederwahlsituation einiger demokratischer Senatsmitglieder höchst unsicher. Und auch für den Senat gilt im Herbst 2022, dass das Ergebnis auf Messers Schneide steht. Sollte einer der beiden Kammern an die Republikaner gehen, dürften Steuererhöhungen politisch nahezu ausgeschlossen sein, was aber bedeuten würde, dass schon die gegenwärtigen Ausgaben nicht solide finanziert wären und die US-amerikanischen Staatsschulden weiter wüchsen. Der linke Enthusiasmus aus Deutschland über das schnelle Durchstarten Bidens und über die Progressivität seines (wirtschafts-)politischen Programms ist mithin aus US-Binnensicht kaum nachzuvollziehen.

Kommen wir nun noch einmal zur Ausgangsfrage etwas grundsätzlicher zurück: Ist Bidens Fiskalpolitik ein Vorbild für Deutschland, wenigstens in einem zielsetzenden Sinne? Die ambitionierten klimapolitischen sowie infrastrukturellen Elemente wird man sich ebenso wie die etwas gelassenere Einstellung zu Staatsschulden zwar auch für Deutschland wünschen, dennoch ist aus Sicht der Ökonomik vor einer allzu großen Fixierung auf fiskalpolitische Vorbilder ganz generell zu warnen. Steuern, Ausgaben und Staatsschulden sind zunächst einmal Instrumente, die weder per se schlecht noch gut sind und die man zustands- und kontextabhängig anwenden sollte. Und dieser makroökonomische Kontext ist ein anderer in Deutschland: Angebotsrestriktionen und damit Inflationsgefahren erscheinen noch etwas weiter entfernt als in den USA; die Geldpolitik ist europäisiert, sodass, anders als in den USA, eine Zinserhöhung, mit der die US-amerikanische Zentralbank auf eine höhere Inflation reagieren müsste, deutlich weniger wahrscheinlich ist; und die Staatsschuldenquote sowie deren Dynamik ist dank der vor der COVID-19-Pandemie sowie irgendwann danach vermutlich wieder bindenden Schuldenbremse weniger problematisch.[4] Um es zusammenzufassen: Wegen ihrer inhärenten Zustands- und Kontextabhängigkeit ist eine deutlich expansivere Fiskalpolitik in Deutschland nach der COVID-19-Pandemie im Vergleich

2 Dabei ist es wichtig zu betonen, dass es um Inflationserwartungen geht. Aktuell tatsächlich etwas höhere Inflationsraten sind eher kein Grund zur Beunruhigung, weil sie sehr stark mit pandemischen Sondereffekten zusammenhängen.

3 Genau genommen sind darunter zwei Senatoren, die keiner Partei angehören, aber in der Regel mit den Demokraten abstimmen.

4 In diesem Sinne ist die deutsche Schuldenbremse, die noch kritisch zu beleuchten sein wird, sogar besser als ihr Ruf. Sie hat im entscheidenden Moment die richtige expansive Fiskalpolitik zugelassen. Allerdings wird sie sich jetzt auch in der Erholungs- und der anstehenden Transformationsphase bewähren müssen.

zum vorherigen Niveau nicht alleine mit dem Hinweis auf die USA und deren aktuell durchaus riskante Fiskalpolitik zu kontern.

Zur gegenwärtigen fiskalpolitischen Diskussion in Deutschland gehört, wie schon vor der COVID-19-Pandemie, die Debatte um die Schuldenbremse (Bachmann und Bayer, 2020). Es gibt dabei zwei Arten des Umgangs mit ihr auf Seiten derjenigen, die sich um eine zu frühe erneute Anwendung der Schuldenbremse und damit um eine zu schnelle Bremsung des Post-COVID-Aufschwungs bzw. um den Erfolg des anstehenden klimapolitischen und infrastrukturellen Transformationsprozesses sorgen: (1) Man schafft es in einem politischen Großkonsens die erneute Anwendung der Schuldenbremse noch ein paar Jahre parlamentarisch hinauszuzögern; (2) oder man ergänzt die Schuldenbremse um eine Investitionsregel in der Verfassung. Beide Ideen sind kritisch zu sehen.

Die erste Vorgehensweise ist mit politischer und verfassungsrechtlicher Unsicherheit behaftet, denn politische Winde können sich drehen und Klagen beim Bundesverfassungsgericht immer eingereicht werden. „Durchwurschteln" ist hier unangemessen. Dies gälte auch für eine verstärkte Nutzung von Finanzierungsvehikeln außerhalb regulärer Haushalte. Aber auch die zweite Vorgehensweise ist letztlich nicht praktikabel. Was ist genau eine Investition? In der volkswirtschaftlichen Gesamtrechnung gibt es zwar eine pragmatische Definition, aber auch die wirft Fragen auf: Warum gelten von privaten Haushalten gekaufte Autos nicht als gesamtwirtschaftlicher Kapitalstock und somit deren Autokäufe nicht als Investitionen, Häuser aber schon? Investitionen in nicht physische Anlagegüter, etwa Software und Patente, werden noch gar nicht so lange zu den Investitionen gezählt. Bei staatlichen Investitionen wird es noch einmal problematischer: Selbst wenn aus einer Staatsausgabe ein physischer Gegenstand resultiert, folgt daraus nicht, dass der für den Staat auch veräußerbar wäre. Warum ein Hörsaalbau eine (gute) staatliche Investition sein soll, das komplementär absolut notwendige Gehalt der darin Lehrenden aber (schlechter) Staatskonsum, ist letztlich nicht nachvollziehbar. Auch an der obigen Diskussion um die Biden-Pläne zeigt sich die Unbrauchbarkeit des Investitionsbegriffs: Die Demokraten sind vermutlich davon überzeugt, auch mit dem American Families Plan eine Investition für Amerika zu tätigen. Warum sollten etwa Sozialausgaben nicht Investitionen in den sozialen Frieden sein? Die ökonomische Theorie entscheidet zwischen Investitions- und Konsumcharakter eines Gutes kontextuell. Eine saubere, für die Wirtschaftspolitik brauchbare und hinreichend kontextlose juristische Definition, die dann auch noch einigermaßen politisch manipulationsfrei ist, erscheint dagegen nicht durchführbar.

Warum nicht über eine unabhängige Schuldenkommission nachdenken? Die Schuldenbremse basiert ja auf der richtigen politökonomischen Einsicht, dass ein sich nicht selbstbindendes Parlament zu sehr Fiskalpolitik für die Gegenwart betreibt. Auf der anderen Seite hat sie mit einer im Prinzip fixen Prozentzahl, die allerdings zustandsabhängig ausgesetzt werden kann, einen schweren Konstruktionsfehler: Sie reagiert nicht auf Marktsignale und ist damit zu starr. Ob Staatsschulden tragfähig sind, hängt wesentlich von den (prognostizierten) Zinsen im Verhältnis zum (prognostizierten) nominalen Wachstum ab. Sind und bleiben die Zinsen niedrig, sind mehr Staatsschulden möglich. Nun haben wir aber in der Geldpolitik sehr gute Erfahrungen damit, marktflexible Regelbindung in die Hände einer unabhängigen Kommission zu legen. Gute Geldpolitik setzt Zinsen in Reaktion auf makroökonomische Bedingungen und Erwartungen. Genau dies könnte eine unabhängige Schuldenkommission, die jedes Jahr die Höhe des Budgetdefizits festlegen würde, auch leisten. Der Rest der Fiskalpolitik, Steuern und Ausgaben, läge wie bisher beim Parlament, nur dass die zusätzliche Nebenbedingung gälte, das vorgegebene Budgetdefizit einzuhalten. Der grundgesetzliche Auftrag einer solchen unabhängigen Schuldenkommission wäre somit, finanzpolitische Spielräume zu eröffnen und die Tragfähigkeit der deutschen Staatsschulden zu gewährleisten.[5] Ihre Mitglieder hätten feste, nicht erneuerbare Amtszeiten. Einen Unterschied zur Geldpolitik gäbe es allerdings: Das Parlament, um es nicht seines Königsrechts zu berauben, könnte die Vorgabe der unabhängigen Schuldenkommission etwa mit einer qualifizierten Mehrheit überstimmen, müsste sich aber in besonderem Maße vor dem Souverän rechtfertigen.

Prof. Dr. Rüdiger Bachmann ist Professor an der University of Notre Dame in den USA.


Literatur

Bachmann, R. und C. Bayer (2020), Her mit den Schulden, Frankfurter Allgemeine Zeitung, 3. Februar.
Congressional Budget Office (2021), The 2021 Long-Term Budget Outlook, März.
